# Implementation of an integrative movement program for residents with dementia in a VA nursing home

**DOI:** 10.1186/s12877-021-02494-2

**Published:** 2021-10-27

**Authors:** Alirameen Akram, Francesca Nicosia, Jennifer Lee, Maria Lee, Lynn Martin, Steven Martinez, Cherry Ordoñez, Michele Woo, Deborah E. Barnes

**Affiliations:** 1 CA Northstate University College of Medicine, Elk Grove, CA USA; 2 University of California, San Francisco, San Francisco, CA USA; 3 9700 West Taron Drive, 95757 Elk Grove, United States CA; 4 San Francisco VA Health Care System, San Francisco, CA USA; 5 Together Senior Health, San Francisco, CA USA; 6 Alliant International University, San Francisco, CA USA; 7 4150 Clement Street, 151R, CA 94121 San Francisco, USA

**Keywords:** Dementia, Nursing homes, Quality of life, Quality of care, Exercise

## Abstract

**Background:**

Preventing Loss of Independence through Exercise (PLIÉ) is an integrative group movement program developed for adults with mild-to-moderate dementia attending day programs. However, many older adults with dementia ultimately require assistance with their activities of daily living and become residents in nursing homes or other long-term care facilities with their complex comorbidities and unique needs. We conducted a post-implementation evaluation of PLIÉ at a San Francisco Veterans Affairs (VA) nursing home to assess reach and effectiveness among residents, staff, and family members who participated in ≥ 1 PLIÉ class from 9/2018 to 6/2019.

**Methods:**

Post-implementation number of classes offered and mean attendance; anonymous satisfaction surveys (5-point Likert scales); qualitative content analysis of open-ended survey responses and clinical progress notes.

**Results:**

Forty-five PLIÉ classes were offered over 9 months. Residents attended an average of 13 ± 12 classes with an average class size of 14 residents, 4 staff members, and 2 family members. Most survey respondents rated the program overall as “very good” or “excellent” (100 % residents, *n* = 15; 87 % staff, *n* = 14; 100 % family members, *n* = 8). Respondents reported improvements in themselves and/or others in four domains: (1) physical, (2) psychological, (3) social, and (4) cognitive. Physical improvements among veterans included mobility, strength, and energy. Psychological improvements included feelings of happiness/well-being, enjoyment, and self-empowerment. Social improvements included connection, social skills, and social support. Cognitive improvements included engagement, communication ability, and focus/attention. Responses were similar among resident, staff, and family member surveys and clinical progress notes. Participants frequently reported improvements in multiple domains (e.g., “The veterans are more alert and engaged, many are smiling and laughing.”). Negative comments were primarily related to logistics, suggesting that the class occur more frequently.

**Conclusions:**

PLIÉ was successfully implemented in a VA nursing home with high attendance and satisfaction among residents, staff, and family members. Participants reported clinically meaningful physical, psychological, social, and cognitive benefits. Other long-term care facilities could potentially benefit from implementing PLIÉ to increase quality of life in residents with dementia.

## Background

By 2050, the prevalence of Alzheimer’s disease and other dementias is projected to increase from 6 to 16 million in the United States, with one million new cases per year [1]. People with dementia (PWD) experience a progressive decline in cognitive function and increasing loss of independence that often negatively impacts their overall well-being, such as their mood and self-image, which can further reduce their quality of life.

Many PWD ultimately require assistance with their basic daily activities and become residents in nursing homes. Overall, 48 % of nursing home residents across the United States have Alzheimer’s or other dementias, and 61 % of PWD in nursing homes have moderate or severe cognitive impairment [2]. Veterans with dementia are especially complex patients with unique needs, since they frequently have mental health comorbidities such as post-traumatic stress disorder, substance use disorders, physical illnesses or injuries such as traumatic brain injuries, and/or may be homeless [3]. Disruptive and challenging behavioral symptoms often prevent the placement of veterans with dementia in community long-term care settings because they may pose health or safety risks to themselves or others, and those with the most severe symptoms are often cared for by nursing homes run by the Department of Veterans Affairs (VA), which are called Community Living Centers (CLCs).

The current mainstay pharmacological treatments for PWD (acetylcholinesterase inhibitors and memantine) are associated with small symptomatic relief for some patients, but ultimately lack neuroprotective effects, do not alter the disease course, and do not improve quality of life [4]. Furthermore, the substantial adverse effects of nausea, vomiting, diarrhea, and fatigue lead many patients to discontinue these medications [5]. In contrast, there is a growing body of evidence that non-pharmacological interventions such as physical activity, cognitive/social stimulation and music therapy can improve a wide array of health outcomes while being much safer and sustainable [6–9]. Yet, few evidence-based non-pharmacological programs have been widely implemented and evaluated, particularly in the VA nursing home setting.

Preventing Loss of Independence through Exercise (PLIÉ) is an integrative group movement program that was originally developed for people with mild-to-moderate dementia attending adult day programs [10, 11]. PLIÉ is grounded in neuroscience and targets abilities that are relatively well-maintained in PWD including the ability to learn new movement sequences to support daily function through repetition and procedural memory; the ability to notice in-the-moment bodily sensations; and the ability to experience joy and connect in meaningful ways with others. PLIÉ is also targets five key domains associated with better quality of life in PWD: physical function, cognitive function, well-being, social connection, and self-esteem [12–17].

**Fig. 1:**
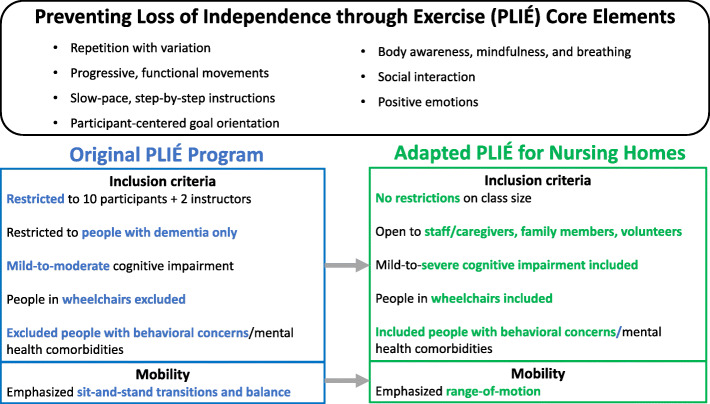
Preventing Loss of Independence through Exercise (PLIÉ) Core Elements and Adaptations

The Core Elements of the PLIÉ program are shown in Fig. 1. Each 45- to 60-minute class begins with participants seated in a circle and the instructor welcoming everyone to create a warm, accepting environment. The initial movement sequences involve tapping, massaging and naming body parts to bring participants into awareness of their bodies in the present moment. This is followed by deep breathing with arm movements and vocalization to promote full body breathing and a mindful rest. The instructor then leads the class through a series of upper and lower body movements sequences that are tailored to the needs and ability levels of the group. Step-by-step instruction and an errorless learning process are used to enable all participants to experience feelings of success. Many movements are interactive to encourage social engagement. Personally meaningful music is incorporated to enhance positive emotions. Each class ends with a repetition of the full-body tapping, massaging, and breathing, and participants are invited to share what brings them feelings of joy, happiness or appreciation.

The original PLIÉ program and an adapted version for PWD and caregivers called Paired PLIÉ have shown clinically meaningful benefits for older adults with dementia and their care partners in the domains of physical, cognitive, and social and emotional functioning [10, 11, 18]. The goal of this study was to adapt PLIÉ for implementation at the San Francisco (SF) VA CLC and to evaluate the program’s impact on residents, staff, and family members.

## Methods

### Adaptation of PLIÉ for VA CLC

In 2017, we received a VA Innovators Award to explore strategies for scaling PLIÉ to reach more veterans. We first conducted semi-structured interviews with key stakeholders, including VA national and local leaders, local clinical providers, community partners, veterans with dementia, and caregivers to learn about the status of VA care for veterans with dementia and unmet needs for CLC residents with dementia. We then identified two CLC clinical champions interested in becoming trained PLIÉ instructors–a Psychiatric Mental Health Clinical Nurse Specialist (LM) and a Geriatric Nurse Practitioner specializing in Restorative Care (ML)–and solicited their input on program adaptations to address local needs. Key adaptations (Fig. 1) included: (1) no restrictions on class size (originally maximum of 10 participants with 2 instructors); (2) allowing participants in wheelchairs (originally excluded) and shifting movement focus from sit-to-stand transition to range-of-motion given greater levels of functional impairment; (3) allowing participants with comorbid mental and physical health issues (originally excluded); and (4) allowing non-resident participants including CLC staff, family or friends, volunteers, and paid caregivers (originally excluded). In addition, we assisted with creating a PLIÉ clinical progress note template so that champions could receive work credit for leading classes and tracking residents’ participation and progress over time.

We then provided clinical champions with 14 weeks of experiential training from February 2018 to June 2018 facilitated by the PLIÉ Senior Instructor (JL) who assisted with study design and implementation. This included: (1) providing a detailed PLIÉ Training Manual; (2) conducting a brief ‘goals assessment’ and medical record review for each potential participant with clinical champions to help tailor the program to best meet participants’ goals and needs; (3) assisting with setting up the room and selecting personally meaningful music for classes; (4) providing and training in use of textured rubber balls for sensory stimulation; (5) providing weekly ‘teaching plans’ describing key program elements that would be emphasized; (6) delivering the program 2 days/week for 12 weeks with clinical champions participating as active assistants; and (7) debriefing with clinical champions after each class to assess what went well, what should be changed, and answer any questions. Feedback from debriefing sessions was continuously incorporated into the PLIÉ program in order to adapt to participants’ evolving needs. After the 14-week training period, the clinical champions led classes on their own for another 12 weeks with the Senior Instructor attending once per week for 4–6 weeks to provide feedback and support. After the clinical champions taught classes on their own, the Senior Instructor returned weekly for 4 weeks and then monthly for 3 months to provide feedback and support.

### Post-implementation evaluation

In June 2019, approximately one year after the initial training was completed, we conducted an evaluation of PLIÉ implementation guided by the first two components of the Reach Effectiveness Adoption Implementation Maintenance (RE-AIM) framework [19, 20]. We assessed reach by reviewing class attendance logs to determine the number of classes offered, number of participants per class, and number of classes attended by each resident. We assessed effectiveness by administering anonymous surveys to those who attended one or more classes and analyzed PLIÉ clinical progress notes of resident participants.

Three related versions of the survey were created for (1) residents, (2) CLC staff and other non-resident participants such as volunteers, paid caregivers, and trainees, and (3) family members/friends. The surveys were administered to residents by the PLIÉ clinical champions who determined cognitive capacity of the residents based on clinical judgement and obtained verbal consent/assent for their participation. The first six questions asked participants to rate specific experiences during PLIÉ classes (e.g., I feel energized; I feel relaxed) using a 4-point Likert scale (never/rarely, sometimes, often, mostly/always). Overall satisfaction with PLIÉ was rated on a 5-point Likert scale (poor, fair, good, very good, excellent). All surveys ended with five open-ended questions about changes observed in themselves and other class participants as a result of participating in PLIÉ classes, what they liked most and least, and other comments.

### Analysis

We analyzed qualitative data using a hybrid deductive and inductive approach to content analysis [21]. First, we applied codes developed in a previous qualitative evaluation of Paired PLIÉ. This included the following domains: physical (e.g., mobility); psychological (e.g., well-being); social (e.g., connectedness); and cognitive (e.g., communication). One coder (AA) applied initial codes to open-ended survey responses. Second, during the coding process, AA identified new concepts from participants’ own words and developed preliminary inductive codes. The larger study team (AA, SM, MW, CO, FN, and DB) regularly reviewed findings and refined new codes through team-based discussions and consensus process. AA also reviewed PLIÉ clinical progress notes to identify descriptions of changes in key domains that were specifically attributed to participation in PLIÉ classes [22].

## Results

### Reach

Clinical champions offered 45 PLIÉ classes during the 9-month evaluation period (September 2018 to June 2019). A total number of 50 residents participated in PLIÉ at least once. On average, residents attended 13 ± 12 classes. The maximum number of classes attended by one resident was 40. In addition to the two clinical champions, PLIÉ classes included an average of 20 participants: 14 residents; 4 staff members, caregivers, volunteers, or trainees (e.g. nursing students, occupational therapy students); and 2 family members or friends of residents. At baseline, residents had a range of cognitive and functional abilities from ambulatory with supervision to non-ambulatory, able to communicate clearly to aphasic, and participating in multiple groups throughout the day and week versus disruptive in other group settings. The outpatient PLIÉ model involved mild-to-moderate cognitive impairment in which the older adults had good physical function (e.g. could stand up and sit down multiple times) whereas our residents were mostly wheelchair bound and needed assistance with transfers to the chair.

### Effectiveness

Sixteen residents, 14 staff, and 8 family members were invited to take the survey, and all but one resident agreed to participate. The vast majority of residents, staff, and family members responded positively to all of the survey items about the PLIÉ classes (Table 1). For example, 100 % of residents, 87 % of staff, and 100 % of family rated the program overall as “very good” or “excellent.” In addition, 100 % of the residents responded positively to statements such as “I feel that I belong,” 93 % of staff responded positively to statements such as “I learn new skills for interacting with residents,” and 100 % of family members responded positively to statements such as “I feel closer to my family member.”

**Table 1:** Satisfaction ratings for residents, staff, and family members

	Percent positive rating (mean ± SD)		
**When participating in the PLIÉ classes**, **to what extent do you experience the following?**	**Residents** ***N = 15***	***Staff N = 14***	***Family N = 8***
**1. I feel that I belong / I learn new skills for interacting with residents / my family member**	100 % (3.9 ± 0.3)	93 % (3.4 ± 0.6)	88 % (3.1 ± 0.6)
**2. I feel that I am accepted / I feel closer to residents / my family member**	100 % (4.0 ± 0.0)	93 % (3.7 ± 0.6)	100 % (3.9 ± 0.4)
**3. I feel that my problems are not unique / I feel that I am part of a team / I feel that the staff here really care about my family member**	100 % (4.0 ± 0.0)	87 % (3.4 ± 1.1)	75 % (4.0 ± 0.0)
**4. I feel energized**	87 % (3.7 ± 0.7)	87 % (3.6 ± 0.8)	88 % (3.5 ± 0.9)
**5. I feel relaxed**	93 % (3.9 ± 0.5)	93 % (3.7 ± 0.6)	100 % (3.6 ± 0.7)
**6. I enjoy being together with a group of people like me / I enjoy my work / I am happy with the care my family member receives here**	100 % (3.9 ± 0.3)	87 % (3.5 ± 0.9)	100 % (4.0 ± 0.0)
**7. How would you rate your overall satisfaction with the PLIÉ program?**	100 % (4.6 ± 0.9)	87 % (4.4 ± 0.9)	100 % (4.9 ± 0.4)

Questions 1–6 rated on 4-point Likert scale (never/rarely, sometimes, often, mostly/always) with responses of often or mostly/always considered positive. Question 7 rated on 5-point Likert scale (poor, fair, good, very good, excellent) with responses of very good/excellent considered positive

### Benefits for residents

Residents, family, and staff also reported benefits in residents during the PLIÉ classes across the following domains: (1) physical, (2) psychological, (3) social, and (4) cognitive (Table 2).

**Table 2:** Changes observed in self as a result of participating in PLIÉ classes

Main Code(s)	Sub-Code(s)	Resident Quote	Staff Quote	Family Quote
Psychological	Well-being	“Happier.”	“It makes me feel good knowing the residents are able to partake.”	“It makes me smile!”
	Self-Empowerment	“I’m not only one who is disabled, some more/some less; I see others more disabled then I try harder.”	“I developed a lot of independence about myself.”	“I’m more willing to exercise.”
Social	Connection / Support	“I feel safe. I feel welcome.”		“I get to know the other veterans.”
	Social Skills	“I have more empathy for others.”	“Learned how to interact with veteran, learned vets’ favorites.”	“I have new skills to interact with my husband as well as other veterans.”
Physical	Energy / Mobility / General	“Improve my physical well-being.”	“Body flexibility.”	
Psychological + Cognitive	Relax + Well-Being + Engagement		“I’m new to them. I like the feeling of calm. And I love seeing veterans so happy and engaged.”	
Psychological + Social	Connection + Enjoy		“Have not attended PLIÉ for a long time but observed vets are interactive and enjoying this activity.”	
	Well-being + Connection		“I noticed that patients are always happy, singing and interacting with each other feels good.”	
Physical + Social	Energy + Connection			“More energy, sense of connection to other vets, staff and community.”
Psychological + Social + Cognitive	Self-Empowerment + Social Skills + Communication/ Language			“I have a new appreciation for life. It’s easier to communicate with my husband. I learn how to communicate with him.”

(1) Physical. Residents reported a variety of personal physical benefits of participating in PLIÉ, including increased energy/alertness, flexibility, mobility, balance, strength, and general physical well-being. For example, a resident reported “[PLIÉ] improves my physical well-being” and a resident noted that “[PLIÉ] gets me out of bed more.” Sometimes, these positive physical changes were coupled with positive social changes as indicated by a family member who reported, “he is more social, and he moves better.”

(2) Psychological. The most common psychological change among residents was improved feelings of well-being and self-empowerment. We defined self-empowerment to be signs of positive thinking, increased motivation towards their goals, and greater self-esteem in taking charge of their life. Examples include, “I’m more willing to exercise,” and “I see people making a greater effort, feeling inspired, feeling connected, feeling hopeful, feeling like [they] belong.” Many residents reported feeling “Happier.” One resident reported feeling motivated by participating in PLIÉ: “I’m not the only one who is disabled. When I see other [residents] who are more or less disabled, I try harder.” Family and staff noticed increased positive emotions and self-empowerment in residents, with some residents also noticing these improvements in other veterans (Table 3). One resident reported, “The others are happier too, especially Mr. X who sings or tries to talk with a smile on his face.”

**Table 3:** Changes observed in others as a result of participating in PLIÉ classes

Main Code(s)	Sub-Code(s)	Resident Quote	Staff Quote	Family Quote
Psychological	Well-Being	“The others are happier too. Especially Mr. X who sings or tries to talk with a smile on his face.”	“I think there’s a lot of improvement about themselves as well.”	“The veterans are more alert and engaged, many are smiling and laughing.”
	Enjoy	“Everyone seemed to enjoy themselves”	“Vets are eager to attend this activity.”	
Cognitive	Engagement		“More engaged.”	“More participation.”
	Focus/ Attention		“The participants are more alert.”	
Physical	Mobility/ Strength	“People are getting stronger.”		
	General	“Yes- it helps everyone do a little more physically.”		
Social	Social Skills		“They are friendly and behave in classes.”	
	Connection		“The dynamic with everybody doing is fantastic.”	
	Social Support		“They get more involved with encouragement from volunteers or facilitators.”	
Psychological + Cognitive	Well-Being + Engagement	“Helps everyone with their mind and spirit. [Other veteran who is usually low-energy] actually participated and was very animated.”		“Veterans look very happy and enthusiastic to participate.”
	Focus/ Attention + Engagement + Well-Being			“The veterans are more alert and engaged, many are smiling and laughing.”
	General + Communication/ Language	“Some made physical gains. Some speak better.”		
Psychological + Social	Self-Empowerment + Connection		“I see people making a greater effort, feeling inspired, feeling connected, feeling hopeful, feeling like you belong.”	
Cognitive + Physical	Engagement + Energy/Activity Level			“Many of the veterans are more engaged and active during and after the class.”
	Communication/Language + General		“Mr. Y can kick the ball and talk!”	
Social + Physical	Social Skills + Mobility/ Strength		“Increased strength, movement, social, and empathy.”	“He’s more social, and he moves better.”
Psychological + Physical + Social	Enjoy + General + Connection			“Enjoyment of movement, play, and interaction.”

(3) Social. Most residents reported increased feelings of connection, social skills, and social support. As one resident mentioned, “The entire group is very friendly and makes me feel safe to express myself. I love the facilitators.” Other comments included, “I have more empathy for others,” and “I feel safe. I feel welcome.” One staff member commented, “I see people making a greater effort, feeling inspired, feeling connected, feeling hopeful, feeling like they belong.”

(4) Cognitive. Several family members observed increased cognitive engagement in their loved ones (e.g., “They are more alert and engaged”). In addition, some residents noticed increased communication/language skills in other veterans. For example, one resident reported “Some [residents] made physical gains. Some speak better,” and another said, “Peer who was not able to speak before is trying to talk more and sometimes does”).

### Confirmation in clinical progress notes

PLIÉ clinical progress notes written by clinical champions after each class echoed changes in physical, psychological, social, and cognitive domains reported by residents, staff, and family members. A total of 419 progress notes were reviewed for 27 residents. Positive changes were noted for 15 residents (56 %). For example, one note described physical improvements: “He demonstrates improved balance. He is now able to transfer with minimal assistance from wheelchair to regular chair.” This same note continued with “He often comments how proud and comfortable he is to be in a regular chair,” indicating related psychological benefits such as self-empowerment. Social and cognitive benefits were reported in another resident, whose progress note stated, “She is more interactive and engaged when she participates in the structured interventions of PLIÉ than when she is out in the general milieu.” Cognitive changes related to communication skills were reported in another resident: “Each week, he practices telling the group what he is grateful for. His ability to make himself understood verbally is increasing with time and practice.” Sometimes evaluations touched on multiple domains: “Mr. G. was fully engaged, enthusiastic, and fully participatory throughout group. This is remarkable for this veteran with advanced dementia. He smiles from ear to ear and demonstrates that he is learning how to use his right hand to work with his paralyzed left hand through the coaching he receives from the clinical champions. In addition, his [caregiver] is learning and modeling how to engage him in ways that are pleasant, meaningful and interesting for him.” Although most progress notes described positive changes, 6 resident notes (22 %) described changes such as falling asleep in class or being asking to leave the session for behavioral disruptions.

### Positive ripple effect of PLIÉ on family and staff

In addition to changes observed in residents, several family members and staff reported experiencing meaningful improvements to their psychological well-being, ability to cope, and caregiving skills resulting from participation in PLIÉ. One family member reported, “I have a new appreciation for life. It’s easier to communicate with my husband. I learned how to communicate with him.” A similar sentiment was shared by a staff member who stated, “I have more empathy. I see more of people’s strengths and resilience and perseverance.” Staff members reported feelings of relaxation and enjoyment (e.g. “I like the feeling of calm. And I love seeing veterans so happy and engaged”). A staff member noticed her greater social connectedness through feeling, “More energy, sense of connection to other vets, staff and community” as a result of participating in PLIÉ.

### Overall satisfaction

When asked what they liked most, majority of participants from all three groups described liking “all” or “everything” about the PLIÉ classes (Table 4**)**. Many participants specifically mentioned liking the social component of the PLIÉ classes. For example, one resident stated, “interactions with others” was his favorite. Others mentioned liking the feelings of well-being derived from the PLIÉ classes, increased self-empowerment, enjoying themselves and seeing others enjoy themselves. For example, another resident stated, “It made me a better person.” Some liked the physical improvements (e.g. “the ability to physically stretch”) and increased energy/activity levels, while some respondents emphasized their joy in seeing participants “light up” and be more cognitively engaged. Some said they loved the music the most. A few described liking two aspects of the program equally (e.g. “I enjoy the music and seeing residents smiling with their family members”).

**Table 4:** What people liked most about PLIÉ

Main Code(s)	Sub-Code(s)	Resident Quote	Staff Quote	Family Quote
Psychological	Enjoy		“Fun.”	“I think most really enjoy it!”
	Well-being	“It made me a better person.”	“Makes patient to feel at home and happy.”	
Cognitive	Engagement		“The participants light up when they get a turn.”	
Social	Social Support	“Gave me the opportunity to try to communicate the frustrations I feel from living here.”	“Seeing vets with physical and cognitive challenges feel they belong and helping each other.”	“Everyone is encouraged to participate to best ability.”
	Social Support	“Encouraging others.”		
	Connection	“Interacting with others.”	“Feeling part of a group.”	
Physical	General	“The ability to physically stretch.”	“Exercises, stretching, breathe.”	
	Energy/Activity Level		“Vets participate even those who sleep at the beginning of the session and begin to actively participate at the beach ball part.”	
Logistics/Others	General	“A lot of merit to it and I try to keep up with it.”	“Loves all of it - somethings that are repeated; other new things.”	“Everything! I like everything.”
	Staff/Volunteers	“The volunteer Ms. M”		“The instructors. They make it fun.”
	Music			“I love the music.”
	Equipment/Technique	“Throwing the ball.”	“Passing around the ball, and upper extremity exercises.”	
Social + Psychological	Social Support + Self-Empowerment	“When the teacher goes around and prompts you to share how you feel at the end of group.”	“Everyone makes an effort and is encouraging.”	
Psychological + Physical	Self-Empowerment + General		“Positive energy and exercise, very beneficial to our residents.”	
Psychological + Logistics/Others + Cognitive	Enjoy + Well-being + Music + Engagement			“I enjoy the music and the sing along I see other family members smiling because they see their family members happy and engaged.”

When asked what they liked least about PLIÉ, several respondents commented on time/frequency, wishing the class was longer in duration, offered more often, or mentioning they disliked when class was canceled. Some staff members mentioned logistics regarding music, such as suggestions to involve more music or trying new music styles. One resident and one staff mentioned the equipment (e.g. “small ball”) as what they liked the least, and one staff member mentioned not having enough staff/volunteers to maximize participation.

## Discussion

 Our post-implementation evaluation of PLIÉ-CLC at SF VA showed that the program reached a large number of veterans, became a popular activity among them, and was effective based on participant-reported benefits in physical, psychological, social and cognitive domains in residents. In addition, PLIÉ-CLC provided a meaningful activity for family members and staff to engage with residents. Several staff and family members reported that they benefited from their involvement in the PLIÉ classes and learned how to better care for residents. It seems likely that improved communication, social skills, understanding, and psychological well-being of these key players further bolstered benefits for residents. Therefore, adapting the original program to include staff and family members may be one of the key changes that contributed to the benefits reported across multiple domains and resulted in long-term program sustainability.

Our results are consistent with studies showing that improving relationships between nursing home staff and residents can increase resident engagement and contribute meaningfully to their quality of life [23]. Working with nursing home residents can be very demanding for staff and lead to burnout, which has negative consequences for all parties involved: residents, family, and staff. Programs that enable staff to feel more empathy and connection with residents may improve their ability to engage residents in appropriate activities, redirect them when needed, and continue supporting them in the most effective ways possible and may ultimately result in greater job satisfaction and less burnout.

Our results also are consistent with studies finding that exercise and nonpharmacological interventions can improve health-related quality of life in nursing home residents with dementia [24, 25]. For example, a randomized control trial of a 12-week high-intensity exercise program for older adults with dementia in nursing homes resulted in a long-term statistically-significant improvement in balance and reduced agitation [26]. Another nursing home study found that movement-oriented restorative care resulted in a more positive self-image after a 12-month intervention compared to the control group [27]. An intervention combining physical with social activity in long-term care facilities found clinically meaningful and statistically-significant improvements in everyday function within a relatively short period of 7 weeks [28]. The strength of PLIÉ-CLC is that it uses a feasible multimodal approach based on neuroscience principles that seems to improve physical, psychological, social and cognitive functions in residents with dementia while additionally supporting the individuals caring for them.

Several staff and family members commented on the importance of incorporating more or different styles of music into the class. This recommendation is supported by studies finding that music therapy significantly reduces depression and agitation in nursing home residents with dementia. Ueda et al.’s systematic review and meta-analysis found that music therapy had moderate effects on anxiety and behavioral symptoms of people with dementia, which was greater when the intervention was longer than 3 months [9]. Playing familiar music has been shown to evoke autobiographical memory, which in turn may improve self-awareness, global cognitive functioning, and neuropsychiatric symptoms [29, 30]. It is postulated that part of music’s ability to enhance coding, memory, and cognition is through modulating physiological responses [29–33]. We observed music to be a powerful tool in stimulating residents collectively, eliciting movements, singing, clapping, and tapping. Integrating music as a modality in movement programs seems to be promising for having positive effects on people with dementia at nursing homes across the country.

One advantage of PLIÉ-CLC is that it is compatible with other CLC programs such as Staff Training in Assisted Living Residences adapted for veterans (STAR-VA), an efficacious interdisciplinary behavioral approach to managing challenging dementia-related behaviors and improving the care residents receive. A key component of STAR-VA is providing “pleasant events” for residents to support their independence, autonomy, and sense of community [34]. Given that veterans expressed feelings of enjoyment in the PLIÉ-CLC classes and residents, family, and staff unanimously supported the program, requesting more classes per week, PLIÉ may provide CLCs and other nursing homes with an efficient model for increasing the frequency of pleasant events.

Although our evaluation results are generally positive, clinical progress notes documented some occurrences of poor engagement, such as residents falling asleep during some sessions or being escorted out of the class because of their disruptive behaviors. These are consistent with common behavioral symptoms in people with dementia and suggest that some residents may be more responsive to PLIÉ than others. It also is possible that evaluation survey responses were more positive than clinical progress notes due to selection or response bias.

Our study’s strengths include collecting both quantitative and qualitative data, administering parallel surveys to residents, staff, and family members with a high response rate, and triangulation of survey responses with clinical progress notes. The consistency of findings using these different methods provide strong support for the validity of our results. Our results corroborate prior PLIÉ studies and the literature that non-pharmacologic interventions are effective for improving many aspects of quality of life in older adults with Alzheimer’s disease and related dementias, including physical function, emotional well-being, social engagement, cognitive function, and self-concept [10, 11, 22]. It builds on this by demonstrating that integrative moment programs can be effective for those with more advanced dementia and in settings beyond adult day programs. Although people living with neurodegenerative diseases such as Alzheimer’s face many challenges, our results suggest that the principles of PLIÉ may help them improve well-being and slow their functional decline. We demonstrated the feasibility of training CLC staff members to deliver PLIÉ classes. In fact, these two clinical champions continued to deliver PLIÉ class two days per week continuously until March 2020, when the COVID-19 pandemic led to cancellation of all group classes. Even during the pandemic, some staff members continued to perform PLIÉ movements 1-on-1 with residents. We are currently remotely training additional CLC staff members in SF and at another facility to deliver PLIÉ classes.

One limitation to our study was that some residents could only participate in one or two sessions because they were residing at the CLC temporarily to provide respite to their caregivers. This may explain the high number of veterans that PLIÉ-CLC reached and likely negatively skewed the number of sessions residents attended on average. There were 9 residents who attended minimal sessions due to respite status. The number of sessions offered in the 9 months also was limited due to a one-month quarantine period due to a norovirus outbreak. Another limitation is that the clinical champions who led the classes administered the evaluation survey to residents, which could have resulted in positive response bias In addition, we did not use validated measures to assess changes associated with PLIÉ, but rather relied on qualitative analysis of participant-reported observations. However, surveys completed by CLC staff and family members were anonymous and yielded results similar to residents, somewhat reducing these concerns.

The primary cost of implementing PLIÉ is related to staff time required to deliver and participate in the training. We estimate that our Senior Instructor dedicated 72 h to this training. This included leading 24 1-hour classes with both clinical champions participating as active assistants and also meeting with them before each class to prepare and after each class to debrief. Clinical champions were able to receive work credit for the classes because they were delivering clinical care to residents who participated. We are currently working to develop a remote training program that would reduce the cost and time burden of the training.

## Conclusions and implications

PLIÉ was successfully adapted and implemented at the San Francisco VA CLC with excellent reach and effectiveness. Satisfaction was high among resident, staff, and family member participants. Participants reported physical, psychological, social, and cognitive benefits even in residents with greater disease severity. The results of our evaluation imply that programs like PLIÉ can be successfully implemented in nursing homes and long-term care facilities to provide a simple, effective model of dementia care that leads to substantial qualitative benefits among multiple domains of health and well-being.

## Data Availability

The datasets used and/or analysed during the current study are available from the corresponding author on reasonable request.

## Abbreviations

CLC: Community Living Center (VA Nursing Home)
PWD: People with Dementia
PLIÉ: Preventing Loss of Independence through Exercise
RE-AIM: Reach, Effectiveness, Adoption, Implementation, and Maintenance
STAR-VA: Staff Training in Assisted Living Residences
SF: San Francisco, California
VA: Veterans Affairs
